# Hospital Expenditure at the End-of-Life: What Are the Impacts of Health Status and Health Risks?

**DOI:** 10.1371/journal.pone.0119035

**Published:** 2015-03-06

**Authors:** Claudia Geue, Paula Lorgelly, James Lewsey, Carole Hart, Andrew Briggs

**Affiliations:** 1 Health Economics & Health Technology Assessment, Institute of Health & Wellbeing, University of Glasgow, 1 Lilybank Gardens, Glasgow, G12 8RZ, United Kingdom; 2 Centre for Health Economics, Building 75, Monash University, Clayton VIC 3800, Australia; 3 Public Health, Institute of Health & Wellbeing, University of Glasgow, 1 Lilybank Gardens, Glasgow, G12 8RZ, United Kingdom; University of Bremen, GERMANY

## Abstract

**Background:**

It is important for health policy and expenditure projections to understand the relationship between age, death and expenditure on health care (HC). Research has shown that older age groups incur lower hospital costs than previously anticipated and that remaining time to death (TTD) was a much stronger indicator for expenditure than age. How health behaviour or risk factors impact on HC utilisation and costs at the end of life is relatively unknown. Smoking and Body Mass Index (BMI) have featured most prominently and mixed findings exist as to the exact nature of this association.

**Methods:**

This paper considers the relationship between TTD, age and expenditure for inpatient care in the last 12 quarters of life; and introduces measures of health status and risks. A longitudinal dataset covering 35 years is utilised, including baseline survey data linked to hospital and death records. The effect of age, TTD and health indicators on expenditure for inpatient care is estimated using a two-part model.

**Results:**

As individuals approach death costs increase. This effect is highly significant (p<0.01) from the last until the 8th quarter before death and influenced by age. Statistically significant effects on costs were found for: smoking status, systolic blood pressure and lung function (FEV1). On average, smokers incurred lower quarterly costs in their last 12 quarters of life than non-smokers (~7%). Participants’ BMI at baseline did show a negative association with probability of HC utilisation however this effect disappeared when costs were estimated.

**Conclusions:**

Health risk measures obtained at baseline provide a good indication of individuals’ probability of needing medical attention later in life and incurring costs, despite the small size of the effect. Utilising a linked dataset, where such measures are available can add substantially to our ability to explain the relationship between TTD and costs.

## Introduction

There is general agreement in the academic literature and amongst policy makers that population ageing alone does not drive healthcare expenditure (HCE) to the extent previously anticipated. Research has found a number of other factors aside from population ageing that explain HCE. One frequently researched factor is remaining time to death (TTD), and studies have concluded that the association between age and HCE actually reflects a stronger relationship between the remaining TTD and HCE, such that TTD is argued to be a better predictor of acute care costs than population ageing per se [1–8]. A limited number of studies have investigated the role that additional factors (such as socio-economic status [9–10]) may play in explaining HCE at the end of life; but few have considered how health behaviour or health risk factors impact on healthcare (HC) service utilisation and HCE at the end of life. A recent study found that health behaviours explained a substantial part of differences in mortality between socio-economic groups [11]; so one could surmise that they might affect costs at the end of life.

Health behaviours or risks have been found to be important predictors of lifetime medical costs. Smoking has long been a focus in the literature and a number of studies have analysed HC costs related to smoking, but have found conflicting results; ranging from findings that show a positive association between smoking and lifetime medical expenditure [12–13] to those that suggest that smokers would not necessarily incur higher costs [14]. With respect to another health risk, Body Mass Index (BMI), previous research revealed a J-shaped association between BMI and HC costs and found a 4% increase in medical costs per unit of BMI increase for BMI >25 [15]. Despite their likely effect on HCE, health behaviours and health risks have not specifically been considered in previous research that has estimated the relationship between population ageing, remaining TTD and HCE. This paper seeks to address this evidence gap and will estimate how health risks and health status measures impact on hospital care costs, the main contributor to overall HC costs, as a population ages and approaches death. The analysis also controls for individuals’ socio-economic status thus providing a comprehensive picture of expenditure profiles at a time that has been identified to be the most expensive.

Many previous analyses of the TTD-ageing-HCE relationship have only included individuals for which a date of death could be observed (decedents); participants without an observed date of death (survivors) are typically right censored and their remaining TTD is unknown. Consequently, any HC costs observed at the end of the study are not necessarily costs incurred at the end of life. This introduces bias and the exclusion of survivors may lead to an overestimation of costs. Such bias is avoided in this paper as survival analysis is employed to predict remaining lifetime for survivors and adjust their observed time before death accordingly [10].

Presented below is a novel but robust analysis of unbiased expenditure for hospital care, controlling for both age and remaining TTD, and health behaviours and health status. The analysis utilises a longitudinal dataset from Scotland where baseline survey data from the 1970s have been linked to subsequent hospital admissions and death records. The breadth of the Scottish data means that explanatory variables, previously not been considered can be included, such as health risk and health status measures at baseline and socio-economic status, thus allowing an examination of the importance of these factors and their impact on expenditure later in life.

## Methods

### Data

This study uses longitudinal data based on a large cohort study undertaken in the 1970s in the West of Scotland. The Renfrew/Paisley study, one of the Midspan studies, covers a period of 35 years [16,17]. The Midspan studies are a series of large occupational and population health surveys carried out in the West of Scotland, starting in the 1960s. The Renfrew/Paisley study includes baseline survey data linked to subsequent acute hospital admissions (Scottish Morbidity Records 01; (SMR01)) and death records. The initial survey took place from 1972–1976 and includes men and women from the towns of Renfrew and Paisley, who were aged between 45 and 64 years at the time of study entry. Participants were asked to complete a questionnaire and also invited to attend screening examinations at clinics. The total dataset includes 15,402 individuals. Detailed information on all Midspan studies can be found here: http://www.gla.ac.uk/researchinstitutes/healthwellbeing/research/publichealth/midspan/.

Permission was given by the Privacy Advisory Committee of the Information Services Division (ISD) to use linked SMR01 data. Ethical approval for this study had been sought and was granted by the University of Glasgow’s ‘Medical Faculty Ethics Committee’(FM01709). All patient records were anonymised prior to the analysis being carried out and no individual patient could be identified.

Linkage was established between the cohort and SMR01 and the National Records of Scotland (NRS), with individuals followed up in terms of hospital use and death until December 2007. SMR01 has episode-based patient records that relate to all acute inpatient and day cases. Care episodes that are excluded from SMR01 are obstetric and psychiatric specialties. Geriatric long stay episodes were part of SMR01 until 1997 but due to this inconsistency and the nature of the care episodes they have been excluded from the analysis. To estimate hospital utilisation multiple inpatient episodes that form an entire hospital stay were summarised into continuous inpatient stays (CIS).

To reduce measurement error, the SMR01 data were checked for data entry anomalies such as duplicate, overlapping and nested episodes, which were discarded from further analysis; in the cohort sample of 15,252 members there were 99,028 CIS. The data were manipulated into 3-monthly quarters for the analytical approach, and after taking account of missing information for the dependent and independent variables, the cohort sample size was 14,860. Costing of hospital episodes was undertaken following the procedure for Healthcare Resource Group (HRG) costing [18] using the quarter of admission as an indicator of when costs were incurred.

### Measures of baseline health status

The questionnaire included questions on smoking behaviour, exercise, home address, bronchitis and angina and clinical data were collected on height, weight, respiratory function (Forced Expiratory Volume (FEV) in 1 second; % predicted FEV1 (derived by dividing actual FEV1 (in litres) through expected FEV1 value, where the expected value is derived from a healthy subset with similar characteristics)), systolic and diastolic blood pressure and cholesterol. Exercise was defined from a question on time spent each day walking to and from work. Smoking status was defined as currently smoking one or more cigarettes/pipes/cigars per day. Data that were subsequently derived include the age at screening and the Carstairs deprivation category based on postcode of residence at screening. The index was developed in 1991 based on the 1981 census as a measure of area deprivation. Category 1 represents the most affluent postcode sectors and category 7 the most deprived ones [19]. Deprivation category was used as the measure of socio-economic status for this analysis. Body-Mass-Index (BMI) was obtained from weight and height.

### Survival analysis

At the end of the study period, death was not observed for all sample members (22% remained alive). Regression analysis was undertaken to estimate the hazard of dying for surviving individuals assuming a Gompertz distribution of the hazard of dying [20]. Time until death was predicted for both survivors and decedents using the following covariates: age at study entry, gender, and socio-economic status. The coefficients obtained from the Gompertz regression were used to calculate the linear predictor of time until failure using covariate values. Based on these results the probability of surviving each year after study entry was calculated using the respective survival function for a Gompertz distribution. This was extended up to t = 100 with the probability of survival becoming infinitesimal. The area under the curve was calculated for that part of the curve that is beyond the censoring date using the trapezoid rule [21]. Predictions of the number of additional years of life for survivors were obtained by adding up values for each of the segments beyond censoring. Predicted additional years of life were transformed into quarters and observed quarters before death adjusted according to the number of additional quarters of life that were predicted. In addition to adjusting observed quarters before death, age at death was adjusted accordingly [10].

### Econometric modelling- explanatory variables

Regression models were employed to estimate the probability of utilising acute inpatient HC services (as measured by CIS) and related costs conditional on positive utilisation for the last 12 quarters of life. The initial exploration of the data showed that costs increased markedly in the last two quarters of life. Exploratory regression analysis determined when TTD became an insignificant predictor for costs. It was therefore decided to analyse the last three years of life, measured in quarters (i.e. 12). The following explanatory variables measuring health status at baseline were included in the regression:

- A dummy variable indicating poor % predicted FEV1 (<70%);
- A dummy variable indicating high systolic blood pressure (SBP) (>140 mmHg)
- A dummy variable indicating high cholesterol levels (> = 6.2mmol/L)
- A dummy variable indicating a high BMI/obesity (>25)
- A dummy variable representing a measure of the time individuals spent each day walking to and from work as a proxy for physical activity (> = 10 minutes)
- A dummy variable representing current smoking status (smoker, i.e. 1 or more cigarettes or pipe/cigars per day).

TTD was included as a series of 12 quarter dummy variables. The quarter furthest away from death (12th quarter) served as the reference category. Age at death (in five year age bands) was measured in seven categories with the youngest age group serving as the reference category. Interactions between TTD in quarters and age at death categories were included to capture any combined effect of ageing and TTD on costs. Gender was included to account for any differences in costs due to gender-specific differences in morbidities with males serving as the reference category. The Carstairs deprivation score was included to account for differences in costs due to socio-economic status. The period of admission in which each quarter before death lies, was included to account for advances in medical technology over time, with the most historic period serving as the reference category.

### Model structure

A two-part model was developed with the first part using a probit link and a binomial distribution to estimate the probability of utilising hospital care in any given quarter before death conditional on a set of regressors X (Equation 1).

$$\text{Pr}(HCE_{i}{}_{,t}>0)=\alpha_{0}+\sum_{a=2}^{7}\alpha_{1}A_{it}+\alpha_{2}S_{i}+\sum_{h=1}^{6}\alpha_{3}H_{i}+\sum_{q=1}^{11}\alpha_{4}Q_{it}+(\sum_{q=1}^{11}\alpha_{5}Q_{i}{}_{t}*\sum_{a=2}^{7}\alpha_{6}A_{it})+\sum_{t=2}^{4}\alpha_{7}Y_{it}+\sum_{d=3}^{7}\alpha_{8}D_{i}+u_{i})$$ Equation (1)

Where: A = age at death categories; S = gender; H = vector of health status and health risk indicators; Q = remaining quarters of life (such that Q*A = interaction of TTD and age); Y = time period dummy indicating the quarter before death; and D = dummy for deprivation category, u_i_ = robust standard errors.

From the second part of the model, estimates of HCE were obtained, conditional on HCE being greater than zero and conditional on the same set of X regressors (Equation 2).

E[HCE]=g(xβ) Equation (2)

with xβ representing the linear predictor for HCE.

The linear predictor of quarterly HCE was estimated fitting a Generalised Linear Model (GLM) clustered on patient identifier. Predicted probabilities of positive HC utilisation, obtained from the first part of the model were multiplied by cost estimates from the second part and average cost estimates were obtained (Equation 3).

E[HCE|X]=Pr(HCE>0|X)×E(HCE|HCE>0,X) Equation (3)

## Results

### Descriptive analysis

Sample characteristics are presented in Table 1. At the end of the study period 22.1% of the sample population were still alive (N = 3,281). The proportion of females in the survivor group was found to be significantly higher than in the decedent group (p<0.01). A higher proportion of individuals living in the most affluent postcode areas were found in the survivor group (p<0.01). A higher proportion of individuals with an increased BMI were observed in the decedent group (55.9%) compared to 52.2% in the survivor group (p<0.01). A significantly higher share of decedents had an SBP above what is regarded as normal (>140mmHg). Significantly more smokers were present in the decedent group and a significantly higher share of decedents had a very low % predicted FEV1 reading (<70%). Survivors were younger than decedents when they entered the study with a mean age of 50.7 years (SD = 4.3) and older at censoring than decedents at death (84.1 years [SD = 4.4] and 74.2 years [SD = 9.3] respectively).

**Table 1:** Sample Characteristics.

Variable	Sample Frequency (%) N = 14,860 (100%)	Decedents Frequency (%) N = 11,579 (77.9%)	Survivors Frequency (%) N = 3,281 (22.1%)	Differences between survivors and decedents (t-test; chi2 test) p-value
Male	6,840 (46%)	5,711 (49.3%)	1,129 (34.4%)	
Female	8,020 (54%)	5,868 (50.7%)	2,152 (65.6%)	p<0.01
Age category at death < 65 years	1,901 (12.8%)	1,901 (16.4%)	0	
Age category at death 65–69 years	1,655 (11.1%)	1,655 (14.3%)	0	
Age category at death 70–74 years	2,098 (14.1%)	2,098 (18.1%)	0	
Age category at death 75–79 years	2,819 (19%)	2,344 (20.2%)	475 (14.5%)	
Age category at death 80–84 years	3,453 (23.2%)	1,988 (17.2%)	1,465 (44.7%)	
Age category at death 85–89 years	2,033 (13.7%)	1,125 (9.7%)	908 (27.7%)	
Age category at death > = 90 years	901 (6.1%)	468 (4%)	433 (13.2%)	Overall p<0.01
Number of HC users	13,300 (89.5%)	2,929 (89.3%)	10,371(89.6%)	
Number of non-users	1,560 (10.5%)	352 (10.7%)	1,208 (10.4%)	p = 0.63
Deprivation Category 1	948 (6.4%)	677 (5.9%)	271 (8.3%)	
Deprivation Category 3	2,008 (13.5%)	1,514 (13.1%)	494 (15.1%)	
Deprivation Category 4	3,234 (21.8%)	2,465 (21.3%)	769 (23.4%)	
Deprivation Category 5	5,381 (36.2%)	4,221 (36.5%)	1,160 (35.4%)	
Deprivation Category 6	2,674 (18.0%)	2,193 (18.9%)	481 (14.7%)	
Deprivation Category 7	615 (4.1%)	509 (4.4%)	106 (3.2%)	Overall p<0.01
BMI < = 25	6,670 (44.9%)	5,103 (44.1%)	1,567 (47.8%)	
BMI > 25	8,190 (55.1%)	6,476 (55.9%)	1,714 (52.2%)	p<0.01
Syst. Blood Pressure <140mmHg	5,824 (39.2%)	4,183 (36.1%)	1,641 (50.0%)	
Syst. Blood Pressure > = 140mmHg	9,036 (60.8%)	7,396 (63.9%)	1,640 (50.0%)	p<0.01
Cholesterol < 6.2mmol/L	7,991 (53.8%)	6,219 (53.7%)	1,772 (54.0%)	
Cholesterol > = 6.2mmol/L	6,869 (46.2%)	5,360 (46.3%)	1,509 (46.0%)	p = 0.76
Walking > = 10 min	11,058 (74.4%)	8,635 (74.6%)	2,423 (73.9%)	
Walking < 10 min	3,802 (25.6%)	2,944 (25.4%)	858 (26.1%)	p = 0.41
Smoker	9,939 (66.9%)	8,130 (70.2%)	1,809 (55.1%)	
Non-Smoker	4,921 (33.1%)	3,449 (29.8%)	1,472 (44.9%)	p<0.01
FEV in 1 sec <70%	2,532 (17.0%)	2,255 (19.5%)	277 (8.4%)	
FEV in 1 sec > = 70%	12,328 (83.0%)	9,324 (80.5%)	3,004 (91.6%)	p<0.01
	Mean (SD)	Mean (SD)	Mean (SD)	
Age at death or Censoring	n/a	74.2 (9.3)	84.1 (4.4)	p<0.01
Age at study entry	54.3 (5.6)	55.3 (5.5)	50.7 (4.3)	p<0.01

*No observations for deprivation category 2.

No significant differences between survivors and decedents were found for two of the health risk and health status measures. The share of the population with a healthy cholesterol level (<6.2mmol/L) in both groups was about 54%. Another non-significant difference between groups was observed for the proxy measure that was used to capture physical activity, i.e. the number of minutes spent each day walking to and from work. In both groups, about 74% spent ten minutes or more walking to or from work each day.

### Survival analysis

Regression results (hazard ratios) for the survival analysis are shown in Table 2. All explanatory variables for time until failure (death) were highly significant and showed the expected sign. On average, male individuals showed a risk of dying that was 63.8% higher than that of female participants. Each additional year of age at study entry increased the risk of dying by 10%. Individuals living in more deprived areas compared to the most affluent areas also had a higher risk of dying with the size of the effect increasing as socio-economic deprivation increases. The ancillary parameter ‘gamma’ is positive, confirming an exponentially increasing hazard of dying as time progresses.

**Table 2:** Results—Gompertz Regression.

Variable	Hazard Ratio	95% CI	
Gender^1^	1.638***	1.096	1.104
Age at Study Entry^2^	1.100***	1.579	1.700
Deprivation Category^3^ = 3	1.172***	1.071	1.284
Deprivation Category = 4	1.226***	1.127	1.336
Deprivation Category = 5	1.314***	1.212	1.425
Deprivation Category = 6	1.148***	1.359	1.615
Deprivation Category = 7	1.733***	1.545	1.945
Gamma	.081***	0.000	0.000
No. of subjects	14,868		
No. of failures	11,587		

*** p<0.01;

^1^Males serve as the reference category;

^2^Age at study entry is measured in years;

^3^Deprivation category 1 serves as the reference category, no observations for deprivation category 2

### Probability of hospital utilisation

In Table 3 (Columns (1) and (2)) results for the probit model are presented. An exponential increase of the probability of accessing hospital care was observed from the penultimate to the last quarter of life.

**Table 3:** Regression Results: Two-part Model.

	Probability of Hospital Utilisation			Cost Ratios		
Column	(1)	(2)		(3)	(4)	
Variable	β	95% CI		β	95% CI	
TTD = 1	1.820***	1.596	2.045	2.027***	1.530	2.685
TTD = 2	0.802***	0.568	1.036	1.973***	1.385	2.810
TTD = 3	0.631***	0.389	0.872	1.757***	1.145	2.695
TTD = 4	0.511***	0.271	0.751	1.809***	1.264	2.590
TTD = 5	0.276**	0.036	0.518	1.604**	1.005	2.560
TTD = 6	0.416***	0.165	0.668	2.064***	1.293	3.295
TTD = 7	0.270*	0.025	0.516	3.188**	1.049	9.684
TTD = 8	0.015	-0.269	0.300	2.684**	1.167	6.170
TTD = 9	0.210	-0.063	0.485	1.376	0.885	2.137
TTD = 10	0.127	-0.137	0.392	0.942	0.654	1.358
TTD = 11	0.137	-0.103	0.377	1.124	0.734	1.723
Age at death 65–69 = (2)	0.195	-0.066	0.458	0.889	0.632	1.250
Age 70–74 = (3)	0.243*	0.001	0.485	1.571**	1.071	2.304
Age 75–79 = (4)	0.313**	0.078	0.548	1.955***	1.378	2.774
Age 80–84 = (5)	0.355***	0.122	0.588	1.963***	1.440	2.676
Age 85–89 = (6)	0.286***	0.093	0.563	1.836***	1.343	2.510
Age > 90 = (7)	0.419***	0.035	0.537	2.487***	1.547	3.998
TTD x Age	Fig. 1			Fig. 2		
Male	0.003	-0.028	0.034	0.849***	0.812	0.888
Deprivation Category = 3	0.082**	0.016	0.149	0.913	0.809	1.031
Deprivation Category = 4	0.078**	0.017	0.141	0.915	0.815	1.027
Deprivation Category = 5	0.088***	0.029	0.148	0.944	0.842	1.059
Deprivation Category = 6	0.054*	-0.009	0.118	1.009	0.894	1.138
Deprivation Category = 7	0.064	-0.018	0.146	0.940	0.814	1.086
Smoker	0.069***	0.038	0.101	0.935***	0.890	0.983
BMI < = 25	-0.064***	-0.092	-0.036	0.986	0.945	1.029
SBP < = 140mmHg	0.058***	0.029	0.087	0.953**	0.914	0.993
FEV1 <70%	-0.014	-0.050	0.021	1.084**	1.016	1.155
Walking > = 10 min	0.003	-0.029	0.035	0.998	0.952	1.047
Cholesterol <6.2mmol/L	0.037**	0.008	0.065	1.014	0.972	1.058
Time period = 1985–1992	0.561***	0.506	0.615	0.880**	0.795	0.974
Time period = 1993–2000	0.875***	0.815	0.936	0.724***	0.651	0.806
Time period = 2001–2007	0.854***	0.888	1.021	0.685***	0.612	0.767
Dead = 1	n/a		n/a	n/a		n/a
Constant	-2.690**	-2.913	-2.468	1877***	1378.082	2558.632
LR Test (TTD*Age)	p<0.001			p<0.001		

*** p<0.01;

**p<0.05,

*p<0.1; Robust standard errors in parentheses;

Deprivation category 1 (most affluent) serves as the reference category;

Age category 1 (<65) serves as the reference category;

TTD = 12 serves as the reference category

**Fig 1:**
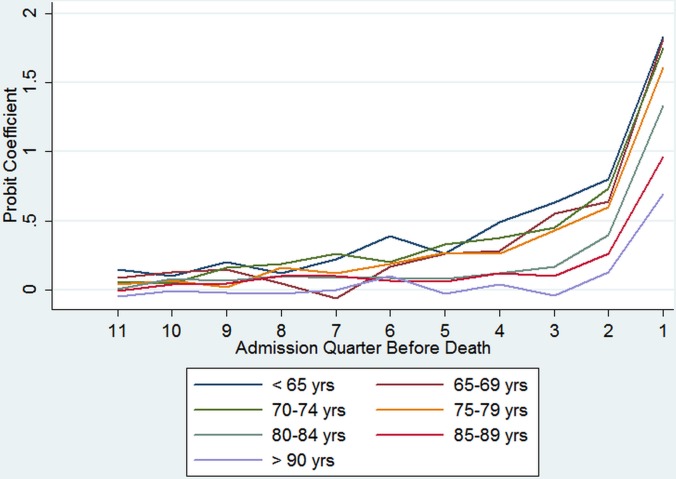
Coefficients for probability of hospitalisation by admission quarter: TTD and age interaction terms.

The age effects relate to the 12th quarter before death. The inclusion of interaction terms allowed age to have a different effect on the probability of being admitted to hospital in each quarter before death. An increased probability of accessing hospital care was observed for individuals aged 70 and older compared to the youngest age group (<65 years). Coefficients for interaction terms between age and TTD, whereby the reference category for age is varied, are shown graphically in Fig. 1. These are presented relative to the 12th quarter before death. A steeper gradient was observed for the younger age groups, especially in the last quarters of life, suggesting a stronger interaction between age and TTD in terms of probability of accessing hospital care for these age groups. Significant interactions between age and TTD can particularly be observed for the last two quarters of life (represented by the steeper gradient).

The coefficient estimates for health status baseline measures revealed that: smokers had a significantly higher probability of being admitted to hospital compared with non-smokers; individuals with a BMI below 25 were less likely to access hospital care than people with a BMI above 25; individuals with a normal SBP (< = 140mmHg) were significantly more likely to access hospital care than people with an SBP of over 140mmHg; and individuals with a cholesterol level below 6.2mmol/L were found to be significantly more likely to access hospital services than people with a level over 6.2mmol/L (although statistically significant, the size of the effect was found to be small). No statistically significant association was found between the probability of being admitted to hospital and the % predicted FEV1, and walking to and from work for more than ten minutes per day. Individuals living in areas with deprivation categories 3, 4 and 5 were significantly more likely to be admitted to hospital compared to individuals from the most affluent areas (category 1). However, this significant effect could not be observed for the two most deprived areas (categories 6 and 7).

### Cost estimates

**Fig 2:**
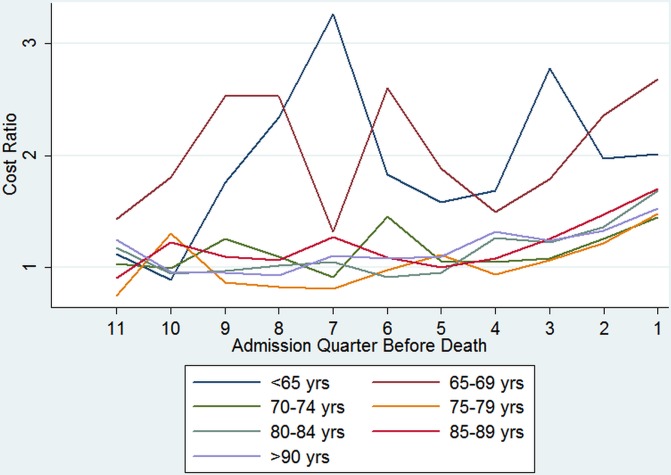
Cost estimates (ratios) by admission quarter: TTD and age interaction terms.

Regression results for the second part of the model, estimating costs given positive utilisation (see Equation 3), are presented in Table 3, columns (3) and (4), again these relate to the youngest age group. Costs were significantly higher in the last eight quarters of life compared with the 12th quarter before death. Age at death was a significant predictor of mean quarterly costs in all age groups, apart from the second youngest age group (65–69 years). Fig. 2 shows interaction effects for each age group plotted against the admission quarter before death relative to the 12th quarter. On average, the two youngest age groups seem to incur higher costs in their last 11 quarters of life compared with the 12th quarter and also compared with the last 11 quarters of life of all other, older age groups. Some of the interaction terms between TTD and age showed a statistically significant association with costs, i.e. the effect on costs of being in a particular quarter before death compared to the 12th quarter also depends on age, however the interaction effect between age and TTD for costs, conditional on having accessed hospital care are less pronounced than the interaction effects for the first modelling part. Costs in the last quarters of life seemed to be much more influenced by age if individuals are younger, as can be seen from the blue and brown lines in the graphs, which show a much more divergent pattern than the lines representing the interactions between TTD and age for the older ages (which are more similar in their variations). These lines also show in the last two quarters before death at least a two-fold increase in costs compared to the 12th quarter.

**Fig 3:**
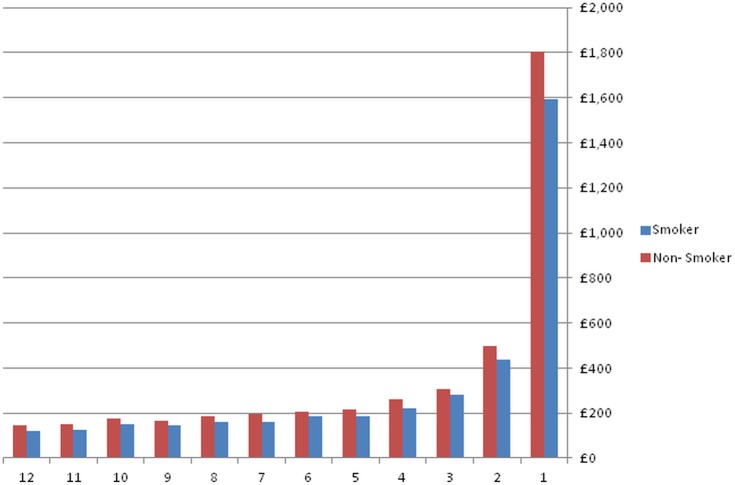
Mean Hospital Costs in GBP (2006/07 prices), comparing smokers and non-smokers.

On average, male individuals incurred significantly lower costs than females (~15%). The effect that the socioeconomic status had on costs, given positive utilisation was very small. Interestingly, it also did not seem to have a statistically significant association with costs. The statistically significant effect of deprivation on the probability of hospitalisation that was observed above was found to disappear. Smokers were found to incur lower costs on average than non-smokers (~7%). Fig. 3 compares quarterly cost estimates for smokers and non-smokers. These are predicted costs that were calculated from Equation 3, which multiplied the first modelling part (probability of accessing hospital services) with the second modelling part (cost estimates, given positive utilisation). Differences in costs can especially be observed in the last quarter before death, where the point estimates for smokers was £1,593 compared to an estimate of £1,806 for non-smokers. Differences in cost estimates are less pronounced the further away from death individuals were.

The effect of BMI was found to be very small and not statistically significant. A SBP of below 140mmHg at baseline led to a significant reduction in costs by about 5%, and a % predicted FEV below 70% led to a significant increase in costs (~8%). No significant effect was observed for the health status indicator of walking to and from work for more than ten minutes a day.

## Discussion

This paper provides an estimate of the independent effect that TTD and age have on expenditure for acute inpatient care. This was done using a longitudinal survey based dataset (Renfrew/Paisley study) linked to acute inpatient records (SMR01) that included previously excluded factors; namely measures of health status and health risks while also controlling for socio-economic status.

Overall, TTD, age at death and the interactions between these two were found to be significant predictors for costs in the last 12 quarters of life thus confirming findings from other national research [2; 5; 22] for a sample of the Scottish population. On average, the two youngest age groups (<65 and 65–69 years) were found to incur higher costs than the older age groups, confirming in part the ‘red herring’ argument put forward by Zweifel and colleagues [2]. However, age was still found to be an important predictor for expenditure and TTD was found to influence costs differently for different age groups, as shown through the inclusion of interactions between TTD and age.

This paper also sought to investigate how health status and health risk measures that were obtained at baseline influenced hospital costs later in life. Although the size of the effect that these variables had on the probability of being admitted to hospital was small, statistical significance could be observed for four of the measures: smoking status, BMI, SBP, and cholesterol at baseline. Notably for SBP and the cholesterol level the converse of what was expected was found: individuals with healthy readings were observed to have a higher probability of accessing hospital care. This might be explained by the ‘worried well’ seeking medical attention earlier and perhaps more frequently. This is confirmed when considering the second part of the model which estimated costs given positive utilisation; the significant effects on the probability of accessing hospital care did not filter through to significantly higher costs being incurred. People from more deprived areas are more likely to reach hospital in the last 12 quarters before death than people from less deprived areas. This might be a reflection of people from more affluent areas being more likely to die at home or in nursing homes compared to people from more deprived areas who might be more likely to die in hospital. This seems to be confirmed when looking at the lack of difference in costs incurred between socio-economic groups, once hospitalised.

The time during which this study was undertaken might provide further explanation as to some of the results. An inverse association between the cholesterol level and the socio-economic status was found in previous research [23–24], i.e. individuals from more affluent areas had a higher reading. The study took place at a time, when public knowledge of the harmful effects of cholesterol was limited. People from more affluent areas could afford to eat red meat and may have consequently had higher cholesterol levels. In turn, people with a healthy cholesterol level may have had unhealthy readings for other health status measures [23–24]. Another possible explanation could be provided through the cut-offs that were chosen for these measures, that is different thresholds might reveal different results. Statistically significant effects on costs were found for the following health indicators: smoking status, SBP, and % predicted FEV1. Of interest is the effect that smoking has on costs. On average, smokers seem to incur lower quarterly costs in their last 12 quarters of life than non-smokers. Findings for the impact that health status and health risk indicators have on costs are very informative, given the time span over which individuals were followed up in terms of their hospital utilisation. Although effects were observed to be small, health status and health risk measures seem to be able to still provide a reasonably good indication of individuals’ probability of needing medical attention later in life (as far as 30 years away) and also of associated costs. This shows that utilising a linked dataset, where such measures can be used in regression modelling can add substantially to our ability of being able to explain the relationship between TTD and costs.

### Limitations and future research

The strength of this study is expressed through the comprehensive linked data with very low attrition rates and minimum missing observations. However like many empirical analyses using longitudinal cohort data it also has limitations. Study participants may have had hospital admissions outside Scotland, i.e. the rest of the UK or even Europe, which are not recorded in the linked Renfrew/Paisley-SMR01 data. Admission quarters have been used to assign costs to a specific quarter before death. This, while used in previous research, does push costs away from death which could lead to an underestimation of costs in the quarters closest to death. As older people tend to be closer to death on average, this may also affect the distribution of costs in the last quarters of life by age category. Further limitations arise from the fact that repeated measurements on health status indicators over the follow-up period were not available and the analyses rely on the assumption that participants had constant health risks throughout. However it does highlight that even baseline health measures taken some decades ago have an impact on future hospital utilisation and costs. Another limitation arises from the fact that only costs for the acute inpatient care sector could be analysed. Although accounting for the main share (close to 60%) of overall HC costs in Scotland [25], it does not provide a complete picture of how population ageing might impact on total expenditure for health and social care. With the ongoing integration of health and social care in Scotland however, future research will be able to include additional data on individuals receiving social care.

Despite these limitations, this study is novel as it provides, for the first time in Scotland, a comprehensive analysis of inpatient care costs, accounting for age, remaining TTD as well as health status and health risk measure. It underpins previous research such as studies on the issue of rationing HC by age; two decades ago Williams put forward the ‘fair innings’ argument, which argued that resources should be devoted to those that would benefit most, i.e. more should be done to enable younger people to survive than should be done to enable older people to survive [26]. This paper provides some understanding of the reality of hospital utilisation, expenditure and possible rationing.

Further contributions to the wider literature include suggestions regarding the relationship between deprivation and costs, although further research to confirm this is required. The analysis identified interesting results for the effect that deprivation category had on the probability of being admitted to hospital and quarterly costs. Once hospitalised, the overall deprivation effect is no longer significant, so while these individuals might be more likely to be admitted to hospital, they do not consequently incur higher costs. Access to HC services and a higher risk of mortality for less affluent areas could serve as an explanation here [27] however this issue certainly requires further research using a representative sample of the population and more refined measures of socio-economic deprivation.
